# Feasibility of Specimen Self-collection in Young Children Undergoing SARS-CoV-2 Surveillance for In-Person Learning

**DOI:** 10.1001/jamanetworkopen.2021.48988

**Published:** 2022-02-17

**Authors:** Jonathan Altamirano, Marcela Lopez, India G. Robinson, Leanne X. Chun, Grace K.-Y. Tam, Nuzhat J. Shaikh, Elisabeth G. Hoyte, Yuan Jin Carrington, Shilpa G. Jani, Elizabeth Y. Toomarian, Julianna C. Hsing, Jasmin Ma, Uma Pulendran, Prasanthi Govindarajan, Andra L. Blomkalns, Benjamin A. Pinsky, C. Jason Wang, Yvonne Maldonado

**Affiliations:** 1 Division of Infectious Diseases, Department of Pediatrics, Stanford University School of Medicine, Stanford, California; 2 Department of Epidemiology and Population Health, Stanford University School of Medicine, Stanford, California; 3 Division of Allergy, Immunology, and Rheumatology, Department of Pediatrics, Stanford University School of Medicine, Stanford, California; 4 Center for Policy, Outcomes, and Prevention, Department of Pediatrics, Stanford University School of Medicine, Stanford, California; 5 Graduate School of Education, Stanford University, Stanford, California; 6 Synapse School, Menlo Park, California; 7 Department of Emergency Medicine, Stanford University School of Medicine, Stanford, California; 8 Department of Pathology, Stanford University School of Medicine, Stanford, California; 9 Department of Health Policy, Stanford University School of Medicine, Stanford, California

## Abstract

**Importance:**

There is an urgent need to assess the feasibility of COVID-19 surveillance measures in educational settings.

**Objective:**

To assess whether young children can feasibly self-collect SARS-CoV-2 samples for surveillance testing over the course of an academic year.

**Design, Setting, and Participants:**

This prospective pilot cohort study was conducted from September 10, 2020, to June 10, 2021, at a K-8 school in San Mateo County, California. The research consisted of quantitative data collection efforts: (1) demographic data collected, (2) student sample self-collection error rates, and (3) student sample self-collection time durations. Students were enrolled in a hybrid learning model, a teaching model in which students were taught in person and online, with students having the option to attend virtually as needed. Data were collected under waiver of consent from students participating in weekly SARS-CoV-2 testing.

**Main Outcomes and Measures:**

Errors over time for self-collection of nasal swabs such as contaminated swabs and inadequate or shallow swabbing; time taken for sample collection.

**Results:**

Of 296 participants, 148 (50.0%) were boys and 148 (50.0%) were girls. A total of 87 participants (29.2%) identified as Asian; 2 (0.6%), Black or African American; 13 (4.4%), Hispanic/Latinx; 103 (34.6%), non-Hispanic White; 87 (29.2%), multiracial; and 6 (2.0%), other. The median school grade was fourth grade. From September 2020 to March 2021, a total of 4203 samples were obtained from 221 students on a weekly basis, while data on error rates were collected. Errors occurred in 2.7% (n = 107; 95% CI, 2.2%-3.2%) of student encounters, with the highest rate occurring on the first day of testing (20 [10.2%]). There was an overall decrease in error rates over time. From April to June 2021, a total of 2021 samples were obtained from 296 students on a weekly basis while data on encounter lengths were collected. Between April and June 2021, 193 encounters were timed. The mean duration of each encounter was 70 seconds (95% CI, 66.4-73.7 seconds).

**Conclusions and Relevance:**

Mastery of self-collected lower nasal swabs is possible for children 5 years and older. Testing duration can be condensed once students gain proficiency in testing procedures. Scalability for larger schools is possible if consideration is given to the resource-intensive nature of the testing and the setting’s weather patterns.

## Key Points

**Question:**

Can school-aged children effectively self-collect lower nasal swabs for COVID-19 surveillance testing within a school environment?

**Findings:**

In this cohort study of 296 school-aged children, participants were able to feasibly self-collect lower nasal swabs for COVID-19 surveillance testing quickly and with few errors.

**Meaning:**

Pediatric self-collected lower nasal swabs are a viable and easily tolerated specimen collection method for SARS-CoV-2 surveillance in school settings, as evidenced by the low error rate and short time window of sample self-collection during testing.

## Teaser

This cohort study assesses whether young children can effectively self-collect SARS-CoV-2 samples for surveillance testing, using parameters of error rates and timing over the course of the 2020-2021 academic year.

## Introduction

The SARS-CoV-2 pandemic has led to more than 51 million documented infections and more than 803 000 deaths in the United States as of December 2021.^1^ Although pediatric cases make up 17.3% of all COVID-19 cases in the United States, schoolchildren should be considered for surveillance for SARS-CoV-2 infection as they can become infected at home or in their communities and may serve as sources of community transmission.^2^ Children younger than 5 years are particularly at risk because they are currently not eligible for vaccination.^3^ Staff working at schools may also be at risk of contracting COVID-19 and should be considered for surveillance as well.

There is an urgent need to assess the feasibility of surveillance measures for SARS-CoV-2 infection in educational settings. According to the US Centers for Disease Control and Prevention, one key component of a comprehensive prevention strategy is to implement vigilant surveillance testing for SARS-CoV-2.^4^ Some school districts have relied on secondary reporting methods (oftentimes from the county or local government) to collect data on transmission after it has already occurred.^5,6^ However, finding an efficient way to test for COVID-19 on-site in schools would allow for schools to identify cases before transmission can occur.

Per UNESCO in March 2021, half of the world’s student population (more than 800 million learners) was still affected by full or partial school closures.^7^ School-aged children have missed out on crucial aspects of in-school learning, including group socialization and access to resources such as special services and nutritional support.^8,9^ Some families of lower socioeconomic status struggled to provide their children with the necessary tools for learning, including school supplies and adequate internet access for online classes.^10,11^ School closures also increased the risk of children falling behind academically and dropping out of school.^12^ While most schools across the country have since reopened, missed class time as a result of illness or isolation still poses a continued threat to students’ learning. Therefore, schools should ensure that they have the proper protocols in place to maintain the safety of staff and students, as well as their household and community contacts.

While rapid SARS-CoV-2 antigen testing at home remains a possible approach to routine surveillance, access and cost of test kits have been a major obstacle. Another possible consideration for SARS-CoV-2 surveillance is school-based testing, and the extent to which children are able to self-collect anterior nasal swabs during school hours without significant disruption to school activities would impact the feasibility of this approach. While previous studies have been able to assess school surveillance methods for up to 2 months, there is a gap in the literature surrounding the feasibility of regular COVID-19 testing in schools over an extended period of time.^5^ We assessed whether young children can effectively self-collect SARS-CoV-2 samples for surveillance testing, using parameters of error rates and timing over the course of the 2020-2021 academic year.

## Methods

This cohort study was conducted at an independent K-8 school in San Mateo County, California, which remained open for on-site learning during the 2020-2021 academic year. The Stanford University School of Medicine’s institutional review board approved this study. The data described here were collected under waiver of consent due to the low risk of the data being collected, on the condition that the individual demographics of a participant would not be tied to their results. Additional study details can be found in Jani et al.^13^ Study data were collected and managed using REDCap (Research Electronic Data Capture) electronic data capture tools hosted at Stanford University School of Medicine.^14,15^ REDCap is a secure, web-based software platform designed to support data capture for research studies, providing (1) an intuitive interface for validated data capture, (2) audit trails for tracking data manipulation and export procedures, (3) automated export procedures for seamless data downloads to common statistical packages, and (4) procedures for data integration and interoperability with external sources. We followed the Strengthening the Reporting of Observational Studies in Epidemiology (STROBE) reporting guideline for methods and results.^16^

The school implemented a hybrid learning model that combined in-person and distance learning opportunities. Classes were taught on-site and broadcast via video teleconferencing (Zoom), and students had the option of attending classes in person or at home in case of an exposure, COVID-19–related symptoms, or a positive COVID-19 test result. Families were also given the option of remaining fully remote until April 2021. Finally, students in grades 7 and 8 were not allowed on campus until October 2020 because their schedules required that they switch classes throughout the day.

### Scheduled Testing

Students were separated into cohorts of 8 to 20 children led by 1 to 2 staff members (cohort leaders) to minimize the risk of schoolwide outbreaks of SARS-CoV-2. Students and staff members who reported on-site to the school were tested weekly for SARS-CoV-2 using pooled reverse transcription polymerase chain reaction (RT-PCR) testing from September 10, 2020, to June 10, 2021.

**Figure 1.**
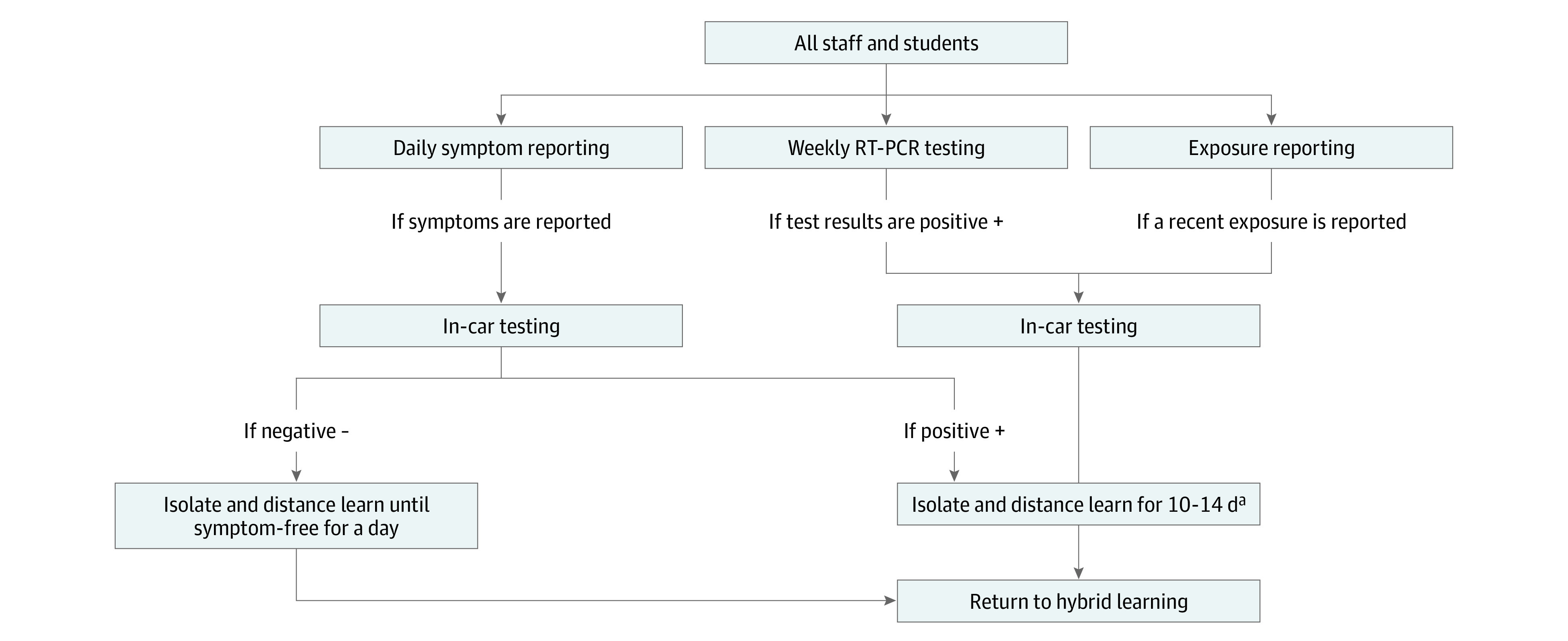
Surveillance Methods Used to Minimize Risk of Transmission of SARS-CoV-2 During On-Site Learning in the 2020-2021 Academic Year ^a^Isolation and distance learning recommendations as a result of either exposure to a confirmed case of SARS-CoV-2 or a positive reverse transcription polymerase chain reaction (RT-PCR) test result changed during the year to reflect the US Centers for Disease Control and Prevention's latest guidelines and recommendations. Isolation period guidelines were shortened to 10 days in December 2020.

On returning from extended school closures, students were brought to school by their caregivers for testing before being allowed back to classes. Regular surveillance testing occurred once a week over 7 hours. Staff and students were also asked to self-report any COVID-19 symptoms prior to coming to campus and were required to wear masks at all times. All students were brought by their cohort leaders to the designated testing site on campus to collect their samples during assigned 15-minute time slots. All testing occurred outdoors, either at the designated testing site or in the school’s parking lot for in-car testing. In-car testing was reserved for individuals who self-reported symptoms or recent exposures to infected individuals. The school’s protocols for how and when staff and students would be tested and the stay-at-home policy implemented based on results can be found in Figure 1. Given the flexibility provided by the hybrid learning model, the number of students tested varied each week. Additional details regarding isolation protocols can be found in eFigure 1 in the Supplement. Students and staff maintained 6-ft distancing while at the testing site, particularly when masks were removed. Clinical research staff donned in full personal protective equipment (PPE) staffed the testing site and observed all sample self-collections. Each week, between 5 and 8 clinical research staff members were present to support testing and between 2 and 4 testing stations were open.

#### Sample Collection Procedure

Anterior nares specimen self-collection was selected for surveillance as opposed to the more invasive nasopharyngeal swab. While the relative sensitivity is lower, 82% to 88% when compared with nasopharyngeal swabs, they are also more easily tolerated during serial testing and decrease the risk of exposure to clinical research staff via nasal secretions during testing.^17^ Clinical research staff provided all students with instructions for anterior nares specimen self-collection and observed them to ensure proper technique. Instructions included making sure not to touch the sterile swab tip (Puritan Sterile Foam Tipped Applicator), inserting the swab fully into one nostril until resistance was felt, and rubbing the swab in 4 circles before repeating the process in the other nostril. Small tabletop mirrors were available at each station to assist with sample collection. Students were also shown a video and instruction sheet (eFigure 2 in the Supplement) prior to their first self-collection.

#### Sample Processing

After anterior nasal swabbing, samples were placed in 3-mL tubes of phosphate buffered saline and submitted to Stanford’s Clinical Virology Lab for US Food and Drug Administration Emergency Use Authorization–approved pooled RT-PCR analysis within 4 hours of specimen collection, using published methods.^18,19^ Stanford Clinical Virology used this methodology to test batches of 4 samples simultaneously. If COVID-19 genetic material was detected in the pooled set of samples, the samples were separated out and rerun through the same process individually to determine the true positives. Parents/guardians and staff members received test results individually 24 to 72 hours after testing and were requested to notify the school in the event of a positive test result. Research personnel also notified the school of cohorts that contained a positive result, but the school was not notified of individual results. While data presented here were collected under waiver of consent, parents/guardians did need to provide written informed consent for their children’s test results to be used for research purposes. These test results, alongside symptom self-report data and health beliefs data, are discussed in Jani et al.^13^

#### In-Car Testing

In-car testing protocols were put into place in the case of reported COVID-19 symptoms, a positive COVID-19 test result, or an exposure to a positive COVID-19 case to limit potential exposures among students and clinical research staff. In-car testing occurred either at Stanford’s Department of Emergency Medicine or in the school’s main parking lot (if symptoms were reported on a testing day). During in-car testing visits, students self-collected anterior nares swabs from within their vehicles while a clinical research staff member provided materials and instructions and observed the self-collection from a distance of 6 ft. In-car testing visits occurred on an as-needed basis and records were not kept on the number of students who tested from their vehicles.

#### Half-Day Testing Description

On the last day of school year testing, the testing schedule was reduced from a full day of testing to a 3-hour half day. Student cohorts arrived to the designated testing site during preassigned 10-minute windows, shortened from 15 minutes and with reduced buffer time between testing windows. On this day, 260 students were tested in 3 hours. In total, 8 clinical research staff members were present and 4 testing stations were open. Four clinical research staff members (M.L., I.G.R., G.K.-Y.T., and E.G.H.) were responsible for observing sample self-collection, 2 (N.J.S. and L.X.C.) were responsible for sanitizing stations in between students, and 2 (J.A. and Y.J.C.) were responsible for processing samples in preparation for submission to our laboratory for RT-PCR testing.

#### Feasibility

Error rates from students’ sample collections were documented from September 2020 to March 2021. Errors were defined as deviations from the standard protocol, such as the use of additional materials or the need for further instruction. Errors were collected by an independent observer who documented the errors committed by blinded clinical research staff members and students. These errors were defined in Table 1. In the event of an error, clinical research staff asked participants to recollect their samples and provided instructions or new materials, as needed. In the specific event of a nosebleed, clinical research staff members provided first aid.

**Table 1.** Types of Errors

Error	Description
Multiple swabs	
Child	More than 1 swab was required to collect a single sample (eg, the swab was dropped, misplaced, or contaminated by the participant).
Coordinator	More than 1 swab was required to collect a single sample (eg, the swab was dropped, misplaced, or contaminated by the coordinator).
Swabbing	
Shallow	The participant failed to insert the swab deep enough into the nasal cavity, leading a staff member to verbally remind the participant of proper procedure.
Inadequate	The participant failed to circle the swab within their nasal cavity enough times, leading a staff member to verbally remind the participant of proper procedure.
Nosebleeds	The participant had a nosebleed during or immediately after sample self-collection.

Only the date and type of error were collected. Demographics of students who committed errors were not documented. Additionally, while errors committed by clinical research staff members were differentiated from errors committed by students, the names of the clinical research staff members who committed these errors were not noted. Lastly, only errors that occurred at the testing tent during student encounters were recorded; errors that occurred during school staff testing or during in-car testing were not documented.

Additionally, an independent observer timed random students’ sample self-collections from April to June 2021. Timing started from the time a student arrived at a testing station to the time they left, including hand and station sanitization, confirmation of identity, sample self-collection, and brief conversation. Clinical research staff members were not made aware of which visits would be timed. All data were summarized using SAS statistical software version 9.4 (SAS Institute).

## Results

### Demographics

Of 296 students in the school, 148 (50.0%) were girls (Table 2). Racial demographics of the student population included Asian (87 [29.2%]), non-Hispanic White (103 [34.6%]), and 2 or more races (ie, Native Hawaiian, other Pacific Islander, American Indian/Alaska Native, or other; 87 [29.2%]). Of those who identified as marginalized racial minorities, 15 (5%) identified as either Black/African American or Hispanic/Latinx and 6 (2.0%) as other. Students were distributed across each K through 8 grade levels, with fifth grade having the most students (n = 39) and eighth grade having the least (n = 27) (Table 2). Median school grade was fourth grade. Demographic information was self-reported.

**Table 2.** Student Demographic Information

Characteristic	No. (%)
Sex	
Male	148 (50.0)
Female	148 (50.0)
Race and ethnicity	
Asian	87 (29.2)
Black or African American	2 (0.6)
Hispanic/Latinx	13 (4.4)
White	103 (34.6)
Other^a^	6 (2.0)
≥2 Race	87 (29.2)
Grade level (n = 296)	
Kindergarten	37 (12.5)
1	30 (10.1)
2	30 (10.1)
3	33 (11.2)
4	31 (10.5)
5	39 (13.2)
6	35 (11.8)
7	34 (11.5)
8	27 (9.1)

^a^ Includes Native Hawaiian, other Pacific Islander, American Indian/Alaska Native, or other.

### Error Rates

**Figure 2.**
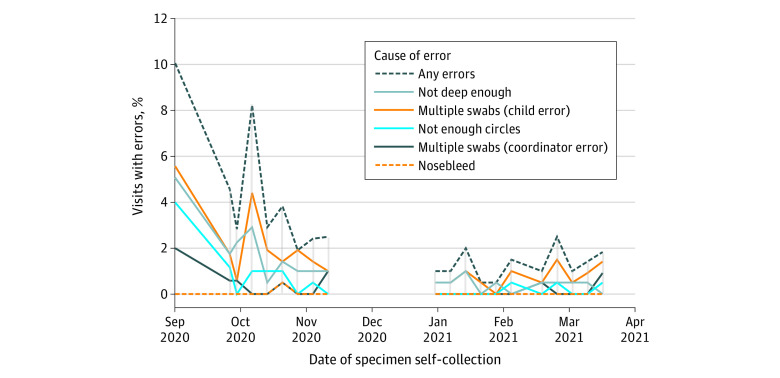
Percent of Errors in Pediatric Specimen Self-collection Over Time Frequency of errors recorded during specimen self-collection from September 2020 to March 2021. Errors occurred in 107 of 4203 student encounters (2.7%), with the highest number of errors occurring on the first day of testing (20 [10.2%]). Type of errors, such as multiple swabs required or shallow swabbing, are graphed separately. The any errors line notes all student encounters with any error on a given week. No specimens were collected from November 23, 2020, to January 3, 2021, coinciding with an extended winter closure implemented by the school study site to minimize transmission of SARS-CoV-2 amid increased community prevalence.

In total, 4203 anterior nares swab samples were collected from 221 students (74.7%) participating in on-site learning from September 2020 through March 2021 when data on error rates were collected. Errors occurred in 2.7% of all student encounters (n = 107; 95% CI, 2.2%-3.2%). Error rates over time are shown in Table 3 and Figure 2, with the highest rate occurring on the first day of testing (20 [10.2%]; 95% CI, 5.9%-14.4%) and the lowest rate in January and February 2021 (1 of 204 [0.5%] for both). Sample collection errors by week never exceeded the first week of testing. Errors decreased to 2.5% by week 3 (n = 5; 95% CI, 0.4%-5.3%). In October 2020, during the fourth week of testing, there was an increase in errors (17 [8.3%]; 95% CI, 4.5%-12.1%) as students in grades 7 and 8 who had been in distance learning joined on-site learning, but errors were decreased again to 2.9% by week 5 (n = 6; 95% CI, 0.6%-5.2%).

**Table 3.** Percentage of Types of Errors Observed in Pediatric Self-specimen Collection Over Time

Test date	No. of tests	No. (%)					
		Visit with errors	Multiple contaminated swabs		Shallow swab collection	Inadequate self-swabbing	Nosebleed
			Child	Clinical staff			
Week 2 of September 2020	197	20 (10.2)	11 (5.6)	4 (2.0)	10 (5.1)	8 (4.1)	0
Week 1 of October 2020	173	8 (4.6)	3 (1.7)	1 (0.6)	3 (1.7)	2 (1.2)	0
Week 1 of October 2020 (second testing)	175	5 (2.9)	1 (0.6)	1 (0.6)	4 (2.3)	0	0
Week 2 of October 2020	204	17 (8.3)	9 (4.4)	0	6 (2.9)	2 (1.0)	0
Week 3 of October 2020	207	6 (2.9)	4 (1.9)	0	1 (0.5)	2 (1.0)	0
Week 4 of October 2020	208	8 (3.8)	3 (1.4)	1 (0.5)	3 (1.4)	2 (1.0)	1 (0.5)
Week 1 of November 2020	206	4 (1.9)	4 (1.9)	0	2 (1.0)	0	0
Week 2 of November 2020	208	5 (2.4)	3 (1.4)	0	2 (1.0)	1 (0.5)	0
Week 3 of November 2020	200	5 (2.5)	2 (1.0)	2 (1.0)	2 (1.0)	0	0
Week 1 of January 2021	209	2 (1.0)	1 (0.5)	0	1 (0.5)	0	0
Week 2 of January 2021	198	2 (1.0)	1 (0.5)	0	1 (0.5)	0	0
Week 3 of January 2021	203	4 (2.0)	2 (1.0)	0	2 (1.0)	0	0
Week 4 of January 2021	202	1 (0.5)	1 (0.5)	0	0	0	0
Week 1 of February 2021	204	1 (0.5)	0	0	1 (0.5)	0	0
Week 2 of February 2021	199	3 (1.5)	2 (0.8)	0	0	1 (0.5)	0
Week 4 of February 2021	194	2 (1.0)	1 (0.4)	1 (0.5)	1 (0.5)	0	0
Week 1 of March 2021	199	5 (2.5)	3 (1.1)	0	1 (0.5)	1 (0.5)	0
Week 2 of March 2021	201	2 (1.0)	1 (0.4)	0	1 (0.5)	0	0
Week 3 of March 2021	215	3 (1.4)	2 (0.7)	0	1 (0.5)	0	0
Week 4 of March 2021	221	4 (1.8)	3 (1.0)	2 (0.9)	0	1 (0.5)	0

Students had an extended winter closure during November and December 2020. Even after 5 weeks without testing, error rates remained low, with errors occurring in less than 1% of student encounters in the first week of testing in 2021 (n = 2). No subsequent weeks in 2021 had more than a 3% error rate during specimen self-collection, indicating that after 6 months of testing, students aged 5 to 14 years were able to successfully self-collect anterior nares samples with minimal errors and retained mastery over the course of breaks.

### Testing Duration

Visits in 2021 for sample self-collection from 296 students occurred during the study period when timing data were collected (April-June 2021). Of those visits, 9.5% (n = 193) were timed. The mean duration of each visit was 70 seconds (95% CI, 66.4-73.7 seconds) On the one half day of testing, an additional 63 of 260 student interactions (24.2%) were timed, with a mean duration of 48 seconds per visit (95% CI, 43.7-52.3 seconds), 22 seconds faster than the 70-second mean across full days of testing.

## Discussion

Our study provided extensive error rate data collected over the course of an entire school year and demonstrated that mastery of self-collected lower nasal swabs is possible for children 5 years and older. As expected when piloting an unfamiliar task, error rates began relatively high in September, around 10% of interactions, but rapidly decreased to 2.9% within 4 weeks. To account for this, we recommend that during the first few weeks of sample collection, schools implementing surveillance testing should dedicate additional time to monitor sample collection and ensure that proper testing procedures are followed. Additional testing materials and PPE should be allotted for the first few weeks of testing to account for the higher rate of materials used during this time.

Although error rates were initially high, they rapidly decreased and stabilized at around 3% within the first month of testing. This shows that students were able to master nasal swabbing techniques and that lower nasal swabbing is well-tolerated by children as young as 5 years. Error rates remained low following a 2-month in-person break in November and December 2020, indicating that mastery is retained even with lapses in testing. The duration of testing was a major strength of our study because in addition to observing mastery over school breaks, it allowed us to see how a pattern of expertise developed among the participants, something that had not been identified previously.

The mean time for testing during the majority of the school year was 70 seconds per participant. Five to 8 clinical research staff members were present each week for testing, with 2 to 4 testing stations open at a time. In June 2021, we piloted a half-day of testing and completed the same number of tests in roughly half the amount of time. During the half day, testing was 48 seconds per participant, pointing to increased efficiency over time as well as the scalability of our testing model. We tackled this accelerated testing model during our final week of testing after a year of testing with consistently low error rates. Our data suggest that schools may feasibly decrease their testing windows over time, after students become more familiarized with anterior nares self-collection. The plateau in error rates suggests that this change in testing could have been implemented once stabilization occurs. Schools should plan to take this learning curve and these mean times into account when constructing a testing schedule.

One consideration to keep in mind during testing is limited personnel and resource availability. To ensure the safety of the students and staff, testing required a large amount of outdoor space to accommodate for 4 appropriately distanced testing stations and 2 tables for supplies. Long hours of testing outside was physically demanding for testing staff members, and periodic breaks were necessary to ensure high-quality testing and adherence to protocol. Additionally, the testing staff required a significant amount of PPE including N95 masks, gloves, hand sanitizer, isolation gowns, and goggles. Even as COVID-19 vaccination rates increase nationwide, schools seeking to implement on-site testing should account for high-demand and back-ordered PPE supplies.

Another consideration is the generalizability of our model in climates that are significantly different from San Mateo County in California. Owing to moderate regional weather patterns, we were able to effectively conduct 7 hours of weekly testing outdoors year-round with minimal interruption from inclement weather. However, this will likely be difficult in regions of the country with more extreme climates. That said, schools should consider setting aside a spacious indoor area, with proper ventilation that allows for social distancing, to be used for indoor testing in the event that outdoor testing is not feasible.

### Limitations

Our study has one key limitation. Keeping the above considerations in mind, our testing model is resource-intensive from both a financial and staffing standpoint. Outside of the materials necessary for testing, RT-PCR assays are costly.^20^ However, the lessons learned even for young children developing mastery in self-collection may be helpful for schools interested in using lower cost tests for surveillance (ie, rapid antigen tests).

## Conclusions

In summary, we found that anterior nasal swabbing is well tolerated and relatively error free among young children, making implementation of on-site testing a feasible way to conduct SARS-CoV-2 surveillance. With time and resource constraints in mind, we believe our model is scalable to larger schools as a way to keep students and teachers safe during in-person learning during these dynamic times. State guidelines, school districts, and budgets should reflect the pressing need for surveillance in schools in light of new SARS-CoV-2 variants and limited vaccine distribution in school-aged children.

## Notes

Supplement. **eFigure 1. **Isolation/Scenario Planning **eFigure 2.** Instruction Sheet

## References

[1] World Health Organization. WHO coronavirus (COVID-19) dashboard. Accessed August 19, 2021. https://covid19.who.int

[2] American Academy of Pediatrics. Children and COVID-19: state-level data report. Accessed August 19, 2021. https://www.aap.org/en/pages/2019-novel-coronavirus-covid-19-infections/children-and-covid-19-state-level-data-report/

[3] Centers for Disease Control and Prevention. COVID-19 vaccines for children and teens. Published February 11, 2020. Accessed August 19, 2021. https://www.cdc.gov/coronavirus/2019-ncov/vaccines/recommendations/adolescents.html

[4] Centers for Disease Control and Prevention. Science brief: transmission of SARS-CoV-2 in K-12 schools and early care and education programs: updated. Published February 11, 2020. Accessed August 19, 2021. https://www.cdc.gov/coronavirus/2019-ncov/science/science-briefs/transmission_k_12_schools.html 34009772

[5] Falk A, Benda A, Falk P, Steffen S, Wallace Z, Høeg TB. COVID-19 cases and transmission in 17 K-12 schools: Wood County, Wisconsin, August 31-November 29, 2020. MMWR Morb Mortal Wkly Rep. 2021;70(4):136-140. doi:10.15585/mmwr.mm7004e3 33507890 PMC7842817

[6] Zimmerman KO, Akinboyo IC, Brookhart MA, ; ABC SCIENCE COLLABORATIVE. Incidence and secondary transmission of SARS-CoV-2 infections in schools. Pediatrics. 2021;147(4):e2020048090. doi:10.1542/peds.2020-048090 33419869 PMC8015158

[7] UNESCO. Education: from disruption to delivery. Published March 4, 2020. Accessed April 12, 2021. https://en.unesco.org/covid19/educationresponse

[8] Jenco M. AAP continues to advocate measures to allow students to return safely to school. *AAP News*. Published online January 5, 2021. Accessed August 19, 2021. https://publications.aap.org/aapnews/news/6597

[9] American Academy of Pediatrics. COVID-19 guidance for safe schools and promotion of in-person learning. Accessed December 23, 2021. https://www.publications.aap.org/pediatrics/article-split/148/3/e2021051438/179735/Preventing-COVID-19-Transmission-in-Education

[10] Sharfstein JM, Morphew CC. The urgency and challenge of opening K-12 schools in the fall of 2020. JAMA. 2020;324(2):133-134. doi:10.1001/jama.2020.10175 32478827

[11] Auxier B, Anderson M. As schools close due to the coronavirus, some U.S. students face a digital ‘homework gap.’ Pew Research Center. Published March 16, 2020. Accessed August 19, 2021. https://www.pewresearch.org/fact-tank/2020/03/16/as-schools-close-due-to-the-coronavirus-some-u-s-students-face-a-digital-homework-gap/

[12] UNESCO. One year into COVID-19 education disruption: where do we stand? Published March 19, 2021. Accessed August 19, 2021. https://en.unesco.org/news/one-year-covid-19-education-disruption-where-do-we-stand

[13] Jani SG, Ma J, Pulendran U, . Prospective pilot study evaluating SARS-CoV-2 transmission-limiting measures in an on-site school. Acad Pediatr. 2021;S1876-2859(21)00617-3. doi:10.1016/j.acap.2021.11.019 34896273 PMC8651529

[14] Harris PA, Taylor R, Thielke R, Payne J, Gonzalez N, Conde JG. Research electronic data capture (REDCap): a metadata-driven methodology and workflow process for providing translational research informatics support. J Biomed Inform. 2009;42(2):377-381. doi:10.1016/j.jbi.2008.08.010 18929686 PMC2700030

[15] Harris PA, Taylor R, Minor BL, ; REDCap Consortium. The REDCap consortium: building an international community of software platform partners. J Biomed Inform. 2019;95:103208. doi:10.1016/j.jbi.2019.103208 31078660 PMC7254481

[16] Vandenbroucke JP, von Elm E, Altman DG, ; STROBE Initiative. Strengthening the Reporting of Observational Studies in Epidemiology (STROBE): explanation and elaboration. Epidemiology. 2007;18(6):805-835. doi:10.1097/EDE.0b013e3181577511 18049195

[17] Zhou Y, O’Leary TJ. Relative sensitivity of anterior nares and nasopharyngeal swabs for initial detection of SARS-CoV-2 in ambulatory patients: rapid review and meta-analysis. PLoS One. 2021;16(7):e0254559. doi:10.1371/journal.pone.0254559 34283845 PMC8291630

[18] US Food and Drug Administration. Stanford Health Care clinical virology laboratory SARS-CoV-2 test EUA summary. Published 2020. Accessed December 20, 2021. https://www.fda.gov/media/136818/download

[19] Hogan CA, Sahoo MK, Huang C, . Comparison of the Panther Fusion and a laboratory-developed test targeting the envelope gene for detection of SARS-CoV-2. J Clin Virol. 2020;127:104383. doi:10.1016/j.jcv.2020.104383 32353760 PMC7195328

[20] Kurani N, Pollitz K, Cotlliar D, Shanosky N, Cox C. COVID-19 test prices and payment policy. Peterson-KFF Health System Tracker. Published April 29, 2021. Accessed August 19, 2021. https://www.healthsystemtracker.org/brief/covid-19-test-prices-and-payment-policy/

