# Supplementary material for: Feasibility of Specimen Self-collection in Young Children Undergoing SARS-CoV-2 Surveillance for In-Person Learning
Source: JAMA Netw Open. 2022 Feb 17;5(2):e2148988. doi: 10.1001/jamanetworkopen.2021.48988 (PMC8855233; doi:10.1001/jamanetworkopen.2021.48988)
Supplement: Supplement. — eFigure 1. Isolation/Scenario Planning eFigure 2. Instruction Sheet [file jamanetwopen-e2148988-s001.pdf]

Supplemental Online Content

Altamirano J, Lopez M, Robinson IG, et al. Feasibility of specimen self-collection in young children undergoing SARS-CoV-2 surveillance for in-person learning. *JAMA Netw Open*. 2022;5(2):e2148988. doi:10.1001/jamanetworkopen.2021.48988

eFigure 1. Isolation/Scenario Planning

eFigure 2. Instruction Sheet

This supplemental material has been provided by the authors to give readers additional information about their work.

© 2022 Altamirano J et al. *JAMA Network Open.*

eFigure 1. Isolation/Scenario Planning

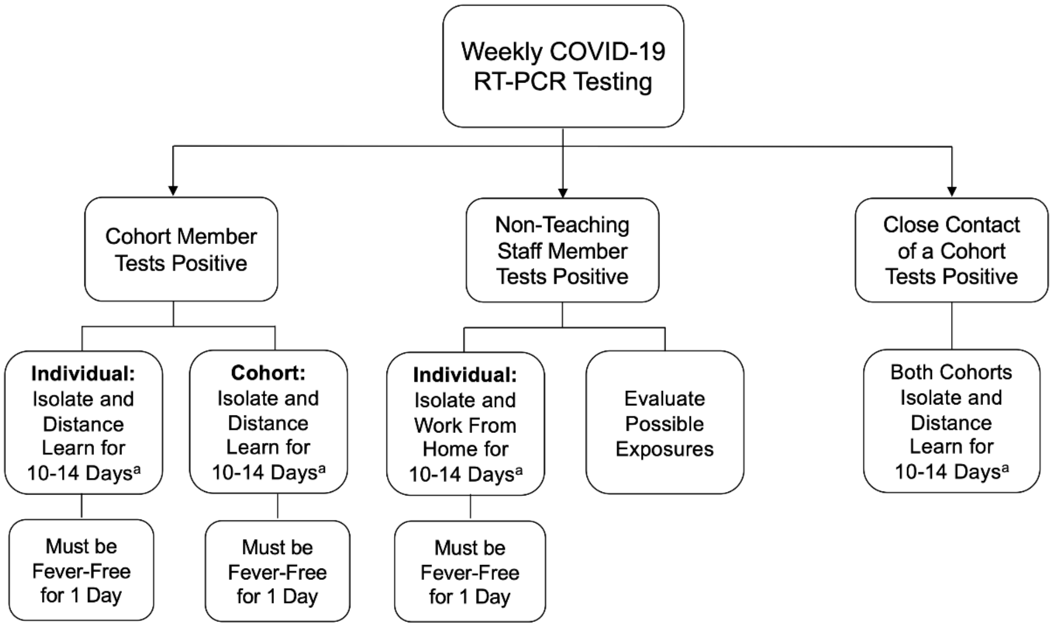

[a]Isolation period after close contact or positive test result changed over the course of the study (from 14 days to 10 days) to reflect the latest guidance provided by the CDC

eFigure 2. Instruction Sheet

© 2022 Altamirano J et al. *JAMA Network Open.*

Swab Instructions

Self-swab nasal specimen collection

We know you aren't feeling well. Thank you for your participation. With your help, we want to understand whether people can test themselves for COVID-19 without assistance from a clinician.

These kinds of tests could one day become available for at-home swabbing for COVID-19 and other diseases like it. We appreciate your time and attention to the instructions below.

1 Open nasal swab

Remove the nasal swab from the wrapper by pulling the two ends of the wrapper apart (like you would to open a band-aid).

Be careful to only touch the handle, not the tip.

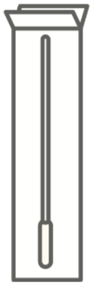

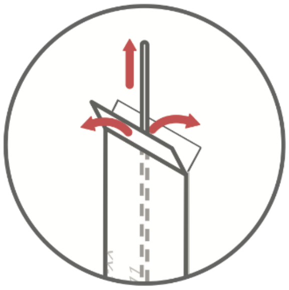

2 Swab nose

Gently insert the **entire** soft tip of the swab into one nostril until you feel a bit of resistance and rub it in a circle around your nostril **4 times.**

Next, gently insert **the same swab** into the other nostril and rub it around the same way.

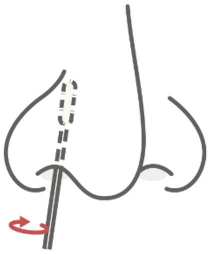

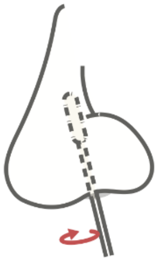

Please watch the provided animation for additional instruction.

3 Put swab in tube

Lower the swab, tip first, into the provided tube.

Once the tip is at the bottom, break the swab handle at the top of the tube by bending back and forth.

Screw the red cap on tightly and hand it to the clinician.

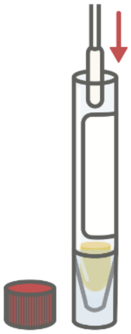

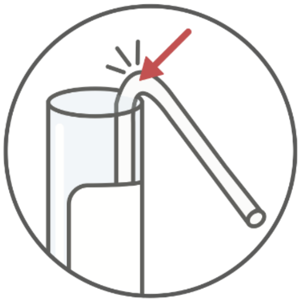

© 2022 Altamirano J et al. *JAMA Network Open.*
